# Different Metrics for Singular Value Optimization in Near-Field Antenna Characterization

**DOI:** 10.3390/s21062122

**Published:** 2021-03-18

**Authors:** Amedeo Capozzoli, Claudio Curcio, Angelo Liseno

**Affiliations:** Dipartimento di Ingegneria Elettrica e delle Tecnologie dell’Informazione, Universitá di Napoli Federico II, via Claudio 21, 80125 Napoli, Italy; clcurcio@unina.it (C.C.); angelo.liseno@unina.it (A.L.)

**Keywords:** near-field/far-field transformations, antenna characterization, singular value optimization, shannon number, mutual information, fisher information

## Abstract

We deal with the use of different metrics in the framework of the Singular Value Optimization (SVO) technique for near-field antenna characterization. SVO extracts the maximum amount of information on an electromagnetic field over a certain domain from field samples on an acquisition domain, with a priori information on the source, e.g., support information. It determines the field sample positions by optimizing a functional featuring the singular value dynamics of the radiation operator and representing a measure of the information collected by the field samples. Here, we discuss in detail and compare the use, in the framework of SVO, of different objective functionals and so of different information measures: Shannon number, mutual information, and Fisher information. The numerical results show that they yield a similar performance.

## 1. Introduction

Extracting the maximum amount of information on an electromagnetic field over a specified domain from field measurements on an acquisition domain, with a priori information on the source, is relevant in a large number of applications.

In optics, we mention the object restoration and image extrapolation problems, in particular, extrapolation outside the pupil from measurements within the pupil [1] as well as image interpolation [2]. In this framework, quantifying the maximum amount of information on a source acquirable from field measurements has been long studied to provide bounds to the information extraction techniques [3,4,5,6,7,8,9,10,11,12,13].

In [3,4,5], the number of significant degrees of freedom of an image is considered as a measure of information and, in [3], its equivalence with the Shannon number is pointed out. The transinformation measure, exploiting the concept of mutual information, is used in [6,7,8,9] to define the information supplied by data samples in a linear imaging system, also accounting for a priori information, which can be then maximized by suitably positioning the acquired samples. The case of both coherent and incoherent imaging has been dealt with in [10]. A formalism for defining, evaluating, and optimizing the degrees of freedom of an optical system has been introduced in [11] and refined in [12]. Finally, the information gained by performing a measurement on a physical system is assessed by the Fisher information in [13].

In microwave and millimeter-wave applications, such problem is of interest for Near-Field/Far-Field (NFFF) [14,15] or Very Near-Field/ Far-Field (V-NFFF) [16] transformations and also for electromagnetic compatibility [17].

To this end, an approach for the fast NF characterization formulating the problem as a linear inverse one has been developed in [14,15,16,17,18]. For such an approach, a linear operator links the source to the field measured over the observation domain so that the source properties can be reconstructed thanks to a proper inversion procedure. When both spectral and spatial support information about the source are available, a reduction, even remarkable, of the number of measurement points can be obtained. In particular, in [14,15], the number and the distribution of the optimal measurement locations are determined by means of the Singular Value Optimization (SVO) procedure [14,15,16,17,18,19], aimed to optimize a proper functional involving the Singular Value Behavior (SVB) of the relevant operator. The effectiveness of the approach has been experimentally validated for several sources and scanning geometries configurations [14,15], leading to a drastic reduction of the number of the measurement samples and of the scanning path length, with respect to conventional as well as optimized approaches. In more detail, in [14], a plane-polar acquisition geometry is used and results over a Ku-band horn antenna are presented while, in [15], “quasi-raster” and plane-polar multi-frequency scannings have been dealt with and a single-ridged broadband horn antenna characterized. The optimality of SVO in terms of capability to reach the same performance of optimal, virtual receiving antennas provided by the application of the Singular Value Decomposition (SVD) approach has been recently shown in [19].

In SVO, the SVB is expressed in terms of a quality parameter. Throughout SVO literature, such a quality parameter has always been expressed in terms of the Shannon number which indicates the amount of information collected through the measured field [3]. Obviously, other ways to measure the information can be adopted in the SVO procedure. Accordingly, it is interesting to compare the performance that different metrics (points of view) have with respect to the SVO approach, a point that is missing throughout the literature.

The novelty of this paper is presenting different metrics for SVO related to different objective functionals handling the SVB of the relevant operator. Besides the Shannon number, mutual information [5,6,7,8,9] and Fisher information [20,21,22] metrics will be adopted in the reported analysis.

For applications other than SVO, different information metrics are indeed often used in preconditioning strategies [23] to improve the convergence properties of quasi-Newton optimizers [24], to quantify the ill-posedness of the reconstruction [25], and to explicitly describe the trade-off between accuracy and resolution [26,27].

The paper is organized as follows. In Section 2, the linear inverse problem dealt with in this paper is introduced in an abstract way for a simplified scalar problem. Section 3 is devoted at first to a short recall of the SVO technique. Later on, the SVO functionals arising from the use of the three differently considered metrics are detailed and discussed. In Section 4, the results are presented. Finally, in Section 5, conclusions follow and future developments are foreseen.

## 2. The Problem

**Figure 1:**
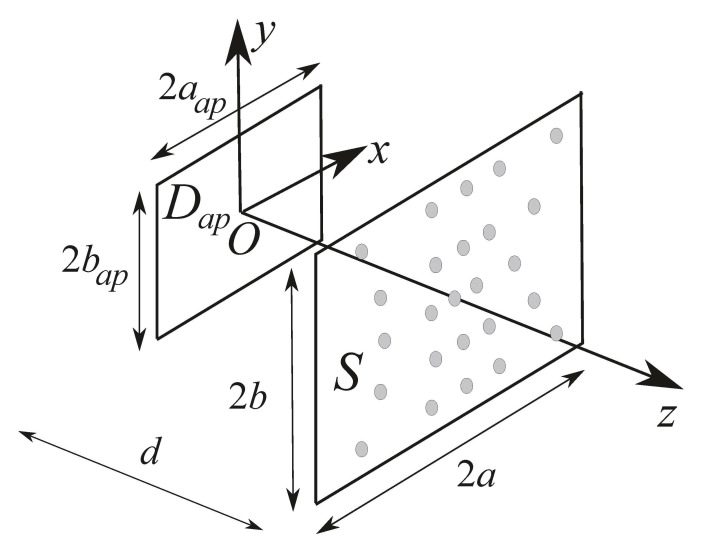
Problem geometry.

Let us consider the problem in Figure 1 depicting a rectangular aperture with effective shape $D_{ap}$, $2a_{ap}\times 2b_{ap}$ sized, located in the z=0 plane of a Cartesian reference system (Oxyz), and centred in *O*. The rectangular aperture represents the Antenna Under Test (AUT).

The radiated field is acquired over a portion *S*, 2a×2b sized, of a NF plane located in front of the source, orthogonal to the *z*-axis, and at a distance *d* from the aperture. A time-harmonic formulation is considered with angular frequency ω, wavelength λ, and propagation constant β=2π/λ.

${\underline{E}}_{a}$ is the (transverse) aperture field and $\underline{E}$ is the transverse field to be measured on *S*. $\underline{E}$ and ${\underline{E}}_{a}$ are linearly related, in an abstract form, as:

(1) $$\underline{E}=A\left({\underline{E}}_{a}\right).$$

We assume an ideal, elementary probe sensing individual field components so that, considering the transverse components only, Equation (1) can be re-written as:

(2) $$E_{x}{\hat{ı}}_{x}=A\left(E_{a_{x}}{\hat{ı}}_{x}\right)\;\;\;\;\;\;\;E_{y}{\hat{ı}}_{y}=A\left(E_{a_{y}}{\hat{ı}}_{y}\right).$$

Accordingly, we can refer to a scalar problem involving just one Cartesian transverse component:

(3) $$E=A(E_{a}).$$

The linear inverse problem we consider is that of recovering $E_{a}$ from *E* by solving Equation (3). Once $E_{a}$ is retrieved, the radiated field and, in particular, the FF can be calculated. For the expression of operator A, the reader is referred, for example, to [15].

## 3. The SVO Approach under Different Perspectives

To solve the problem at hand, two points must be addressed: A discretization strategy making the numerical solution affordable and the ill-conditioning issue.

### 3.1. Discretization

To get a discrete version of the problem in Equation (3), as a first step we need to evaluate it at a finite number, say *N*, of observation points ${\underline{r}}_{1},\ldots ,{\underline{r}}_{N}$. As a second step, we need to describe the aperture field by a finite number of unknown parameters. To this end, let us observe that, since both $E_{a}$ and its Plane Wave Spectrum (PWS) have bounded support, the relevant $E_{a}$ belongs to the finite *M*-dimensional space spanned by Prolate Spheroidal Wave Functions (PSWFs) $\phi_{m}$, m=1,…,M, with a proper space-bandwidth product [14,15,16,17,18], namely:

(4) $$E_{a}\left(x,y\right)=\sum_{m=1}^{M}i_{m}\phi_{m}\left(x,y\right).$$

Details on the representation (4) can be found in [28]. Determining $E_{a}$ thus amounts to determine the vector $\underline{i}=\left(i_{1},\ldots ,i_{M}\right)$.

By sampling the radiated field at ${\underline{r}}_{1},\ldots ,{\underline{r}}_{N}$ and accounting for (4), Equation (3) can be, then, discretized as follows:

(5) $$\underline{\underline{Z}}\left({\underline{r}}_{1},\ldots ,{\underline{r}}_{N}\right)\;\underline{i}=\underline{v},$$

where $\underline{v}$ is the vector containing the *N* measured field samples and the matrix $\underline{\underline{Z}}$ is given by:

(6) $$Z_{nm}=<A\left(\phi_{m}\right),\delta \left(\underline{r}-{\underline{r}}_{n}\right)>.$$

The determination of the unknown vector $\underline{i}$ does amount at the inversion of the linear system of Equations (5). The problem of inverting Equation (5) is ill-conditioned, so ill-conditioning is the next point to be faced.

### 3.2. Ill-Conditioning

To face ill-conditioning, the “complete” reconstruction of the unknowns $\underline{i}$ must be dismissed and a regularization method must be adopted, e.g., the Truncated SVD (TSVD) or the L-curve strategy [29], to retrieve only the information robustly contained in the data. Furthermore, even though the inverse problem to be solved is linear, a number of data larger than that of the unknowns is required.

However, before applying regularization, the following question arises: Which is the most convenient sampling point distribution from the ill-conditioning point of view? In other words, are all the possible distributions of the sampling points equivalent, or it is possible to select a NF measurement grid able to improve the SVB? Indeed, the SVs of $\underline{\underline{Z}}$, say $\left\{\sigma_{1},\sigma_{2},\ldots ,\sigma_{M}\right\}$, can be properly tuned by changing the number and locations of the sampling points. Henceforth, we will assume the $\sigma_{m}$’s ordered in a decreasing way. In particular, to mitigate the ill-conditioning, the number and the spatial distribution of the NF samples should be chosen as the ones optimizing a functional, say $\Phi (\sigma_{1},\sigma_{2},\ldots ,\sigma_{M})$, expressing the degree of conditioning of $\underline{\underline{Z}}$. This is a way of preconditioning [23], or pre-filtering, or a first form of regularization consisting of shaping the spectrum (SVB) of $\underline{\underline{Z}}$ and that we have called SVO. The quality parameter of the SVB depends on the definition of Φ, but we expect that different quality parameters, if coherently defined, lead to similar results, apart from optimization issues.

It should be finally noticed that the ill-conditioning concept is intimately related to that of the amount of collected information [30]. Accordingly, ill-conditioning can be mitigated by improving the amount of information acquired by the data. In the following, three measures of information will be detailed.

### 3.3. Condition Number

A first possible definition of Φ is through the condition number of $\underline{\underline{Z}}$. As known, the condition number is the ratio between the largest and smallest considered SVs $\sigma_{1}/\sigma_{r}$, where *r* is the number of vector components of the unknowns retained during the inversion. Anyway, what is observed, also by a numerical analysis not shown here for the sake of brevity, is that $\sigma_{r}$ is often not much responsive on the sampling locations ${\underline{r}}_{n}$’s, which is opposite to what happens to the larger SVs. In other words, an optimization procedure is typically not able to effectively improve the smallest singular value $\sigma_{r}$, leading to a very poor dynamic. This makes the use of the condition number in the definition of the objective functional unreliable to improve the SVB. Fortunately, it is possible to improve singular values not considered in the merit figure $\sigma_{1}/\sigma_{r}$. This enables the use of the below detailed metrics.

### 3.4. Shannon Number

To achieve a satisfactory SVB, also improving the degree of conditioning, the following functional, assuming N≥M, has been considered up to now in SVO [14,15,16,17,18]:

(7) $$\Phi^{\left(1\right)}\left({\underline{r}}_{1},\ldots ,{\underline{r}}_{N}\right)=\sum_{m=1}^{M}\frac{\sigma_{m}\left({\underline{r}}_{1},\ldots ,{\underline{r}}_{N}\right)}{\sigma_{1}\left({\underline{r}}_{1},\ldots ,{\underline{r}}_{N}\right)}.$$

Functional $\Phi^{\left(1\right)}$ should be maximized to obtain the sampling. Details on the maximization of $\Phi^{\left(1\right)}$ can be found, for example, in [18].

To interpret its meaning, let us first observe that, at the numerator, the sum of the SVs $\sum_{m=1}^{M}\sigma_{m}$ is the Shannon number [3]. The subsequent normalization by $\sigma_{1}$ can be easily explained as follows. For the applications of interest, the $\sigma_{m}$’s exhibit a step-like behavior. In other words, they are approximately constant up to a certain index $m_{0}$ after which they suddenly fall to zero. Exasperating such a behavior, let us assume for a moment that, up to $m_{0}$, the SVs are all equal, and, then, beyond $m_{0}$, they all vanish. In this case, the normalized sum would furnish the number of non-zero SVs. In the actual case when the $\sigma_{m}$’s do not exhibit such a sharp behavior and generalizing the expounded extreme case, the functional (7) can be interpreted as a measure of the relevant SVs. Here, by relevant SVs, we mean all those SVs retained by the TSVD, namely, those whose square amplitude is larger than the noise level. This corresponds to the (weighted) dimension of the vector space of the unknowns that can be actually reconstructed from the data [11,12].

### 3.5. Mutual Information

Using the SVD of $\underline{\underline{Z}}$, the unknown $\underline{i}$ can be expressed as:

(8) $$\underline{i}=\sum_{m=1}^{M}\alpha_{m}{\underline{\psi }}_{m}^{\left(R\right)},$$

where $\psi_{m}^{\left(R\right)}$, m=1,…,M, are the right singular vectors. Accordingly, the actual unknowns become the $\alpha_{m}$’s.

By using the SVD expansion, the measured field can be written as:

(9) $$\underline{v}=\sum_{m=1}^{M}\sigma_{m}\alpha_{m}{\underline{\psi }}_{m}^{\left(L\right)},$$

where the ${\underline{\psi }}_{m}^{\left(L\right)}=\left(\psi_{1}^{\left(L\right)},\psi_{2}^{\left(L\right)},\ldots ,\psi_{n}^{\left(L\right)},\ldots ,\psi_{N}^{\left(L\right)}\right)$’s are the left singular vectors of $\underline{\underline{Z}}$.

The presence of additive noise leads, in the first place, changes Equation (9) into:

(10) $$\underline{v}=\sum_{m=1}^{M}\sigma_{m}\alpha_{m}{\underline{\psi }}_{m}^{\left(L\right)}+\underline{w},$$

where $\underline{v}=\left(v_{1},v_{2},\ldots ,v_{n},\ldots ,v_{N}\right)$ and $\underline{w}=\left(w_{1},w_{2},\ldots ,w_{n},\ldots ,w_{N}\right)$ is the noise vector. Obviously, the noise corrupting the data, depending on its source, can have different statistics and can be differently modeled. Here, we are assuming additive noise.

Let us denote by $p_{u}\left(\underline{\alpha }\right)$ the Probability Distribution Function (PDF) of the unknown $\underline{\alpha }$, with $\underline{\alpha }=\left(\alpha_{1},\ldots ,\alpha_{M}\right)$, and by $p_{d|u}\left(\underline{v}|\underline{\alpha }\right)$ the conditional PDF of the data, given the unknown. From such PDFs, we can obtain the unconditional PDF of the data, namely, $p_{d}\left(\underline{v}\right)$, and the a posteriori PDF of the unknown, namely, $p_{u|d}\left(\underline{\alpha }|\underline{v}\right)$. The quantity $p_{u|d}$ expresses the state of knowledge on the unknown $\underline{\alpha }$, bearing in mind the observed data $\underline{v}$.

Mutual information is provided by [6]:

(11) $$I=\int p_{u,d}\left(\underline{\alpha },\underline{v}\right)\log_{2}\left\{\frac{p_{u|d}\left(\underline{\alpha }|\underline{v}\right)}{p_{u}\left(\underline{\alpha }\right)}\right\}d\underline{\alpha }d\underline{v}.$$

In order to determine a manageable closed-form expression for (11), statistical models for the noise and unknown aperture field are needed. A Gaussian model for the noise is easily acceptable, leading to:

(12) $$p_{d|u}\left(\underline{v}|\underline{\alpha }\right)=\frac{1}{\sqrt{{\left(2\pi \right)}^{N}det\left(\underline{\underline{W}}\right)}}e^{-\frac{1}{2}{\left(\underline{v}-\underline{\underline{Z}}\;\underline{\alpha }\right)}^{†}{\underline{\underline{W}}}^{-1}\left(\underline{v}-\underline{\underline{Z}}\;\underline{\alpha }\right)},$$

concerning the unknown, in absence of a priori information, a uniform statistical distribution of the coefficients of the aperture field would be the most natural choice. Such an assumption is typically exploited to provide a statistical interpretation of TSVD in absence of a priori information, see [31]. However, it should be noticed that, by proper minimization of divergence measures, for example, the Kullback–Leibler divergence [32], a Gaussian distribution can approximate, within certain limits, a uniform one. Consequently, we will assume both $p_{u}$ Gaussian, namely and:

(13) $$p_{u}\left(\underline{\alpha }\right)=\frac{1}{\sqrt{{\left(2\pi \right)}^{M}det\left(\underline{\underline{A}}\right)}}e^{-\frac{1}{2}{\underline{\alpha }}^{†}{\underline{\underline{A}}}^{-1}\underline{\alpha }},$$

where $\underline{\underline{A}}$ and $\underline{\underline{W}}$ are the covariance matrices of the unknown and of the noise, respectively. According to the above hypotheses, the mutual information *I* can be written as [6,7,8]:

(14) $$I=0.5\log_{2}\left\{\frac{det(\underline{\underline{Z}}\;\underline{\underline{A}}\;{\underline{\underline{Z}}}^{†}+\underline{\underline{W}})}{det(\underline{\underline{W}})}\right\}.$$

Under the same hypotheses as in [6,7,8,9], $\underline{\underline{A}}$ and $\underline{\underline{W}}$ can be written as $\underline{\underline{A}}=\sigma_{U}^{2}\underline{\underline{I}}$ and $\underline{\underline{W}}=\sigma_{W}^{2}\underline{\underline{I}}$. Accordingly, Equation (14) becomes:

(15) $$I=0.5\log_{2}\left\{\frac{det(\sigma_{U}^{2}\underline{\underline{Z}}\;{\underline{\underline{Z}}}^{†}+\sigma_{W}^{2}\underline{\underline{I}})}{\sigma_{W}^{2}}\right\}$$

which readily rewrites as:

(16) $$I=0.5\sum_{m=1}^{M}\log_{2}\left\{\frac{\sigma_{U}^{2}}{\sigma_{W}^{2}}\sigma_{m}^{2}+1\right\}.$$

The expression of the mutual information by Equation (16) has a meaningful interpretation. Indeed, $\sigma_{U}^{2}\sigma_{m}^{2}$ represents the power of the *m*-th vector component of the data, on the SVD basis, while $\sigma_{W}^{2}$ represents the power of the noise superimposed to the same vector component [11,12]. Accordingly, $0.5\log_{2}\left\{\frac{\sigma_{U}^{2}}{\sigma_{W}^{2}}\sigma_{m}^{2}+1\right\}$ can be interpreted as the contribution to *I* gathered from the *m*-th vector component of the unknown when measuring the corresponding vector component of the data [9], and the information sums up thanks to the exploited hypotheses. Thus, whenever $\sigma_{U}^{2}\sigma_{m}^{2}\ll \sigma_{W}^{2}$, the information associated to the measurement of the *m*-th component is approximately $0.5\log_{2}\left\{\delta +1\right\}$, with δ is a value approaching 0, and thus vanishing. This occurs since the corresponding datum is corrupted by the noise and, when applying the regularization strategy, this component should be filtered out, for example, by a Truncated SVD (TSVD). Therefore, to avoid such a filtering, δ should be made as large as possible. This corresponds to the idea, already exploited in the Shannon number case, to increase the $\sigma_{m}$’s as much as possible. This purpose can be pursued by maximizing the mutual information, *I*.

We stress that the above assumption of uniform statistical distribution of the coefficients of the aperture field and its Gaussian distribution approximation tackle the case when no a priori information is available on the aperture field distribution apart from the rectangular shape and dimensions. In the case when a priori information is available, the above formulation should be updated accordingly and the performance of SVO changes.

Nevertheless, a normalization of the mutual information is convenient, in the same fashion as for the Shannon number case, to make the functional express a measure of the (weighted) dimensionality of the actually reconstructable unknown space. For the considered case of mutual information, this can be done by considering the ratio of the contribution gathered by measuring the *M* vector components of the data and that gathered by the measurement of most relevant component, in particular, that associated to the largest singular value (SV) [7]:

(17) $$D=\frac{\sum_{m=1}^{M}\log_{2}\left\{\frac{\sigma_{U}^{2}}{\sigma_{W}^{2}}\sigma_{m}^{2}+1\right\}}{\log_{2}\left\{\frac{\sigma_{U}^{2}}{\sigma_{W}^{2}}\sigma_{1}^{2}+1\right\}}.$$

Obviously, the dimensionality *D* depends on the NF sampling points, which can be then chosen to maximize it. Accordingly, using mutual information leads to the optimization of the following functional:

(18) $$\Phi^{\left(2\right)}\left({\underline{r}}_{1},\ldots ,{\underline{r}}_{N}\right)=\frac{\sum_{m=1}^{M}\log_{2}\left\{\frac{\sigma_{U}^{2}}{\sigma_{W}^{2}}\sigma_{m}{\left({\underline{r}}_{1},\ldots ,{\underline{r}}_{1}\right)}^{2}+1\right\}}{\log_{2}\left\{\frac{\sigma_{U}^{2}}{\sigma_{W}^{2}}\sigma_{1}{\left({\underline{r}}_{1},\ldots ,{\underline{r}}_{1}\right)}^{2}+1\right\}}.$$

Note that the rationale for optimizing functional $\Phi^{\left(2\right)}\left({\underline{r}}_{1},\ldots ,{\underline{r}}_{N}\right)$ is essentially the same as that for optimizing functional $\Phi^{\left(1\right)}\left({\underline{r}}_{1},\ldots ,{\underline{r}}_{N}\right)$.

### 3.6. Fisher Information

The problem of optimally placing sensors using the Fisher Information Matrix (FIM) has already been faced throughout the literature. In particular, in [20,21,22], the maximization of the determinant of the FIM has been used to rank potential sensor locations for on-orbit modal testing. Indeed, sensor locations providing dependent information which contributes to lower the value of such a determinant, should receive lower rank and should be eventually deleted. Moreover, in [27], the maximization of the determinant of the FIM has been used to face the problem of optimizing the measurement locations in antenna near-field characterization and in particular to determine a probability measure where allocating the probe, once the overall number of measurements is prefixed. Therefore, we test the use of the Fisher information also in SVO.

Accordingly, under the same hypotheses of the previous Section, we here introduce the FIM $\underline{\underline{I}}$ whose generic element $I_{kl}$ is equal to [27,33]:

(19) $$I_{kl}=\frac{1}{\sigma_{W}^{2}}\sum_{n=1}^{N}\frac{\partial v_{n}}{\partial \alpha_{k}}\frac{\partial v_{n}^{*}}{\partial \alpha_{l}^{*}}.$$

By using the SVD expansion (9), the $I_{kl}$’s can be expressed as:

(20) $$I_{kl}=\frac{\sigma_{k}\sigma_{l}}{\sigma_{W}^{2}}\sum_{n=1}^{N}\psi_{k_{n}}^{\left(L\right)}\psi_{l_{n}}^{(L)*}.$$

On resorting to the orthonormality of the ${\underline{\psi }}_{k}^{\left(L\right)}$’s, then:

(21) $$I_{kl}=\frac{\sigma_{k}^{2}}{\sigma_{W}^{2}}\delta \left(k-l\right).$$

with δ(k−l) as the Kronecker symbol.

The introduction of the FIM enables expressing the Cramér–Rao bounds for estimating the *k*-th component of $E_{a}$ by an unbiased estimator [34]. Following [34], the larger the singular values of the FIM, the more accurate the reconstructions.

According to the above, in this paper, we consider the maximization of the determinant of the FIM as a further possibility to determine the sampling locations. Being the determinant of the FIM related to the product of the $\sigma_{m}^{2}$’s, then the functional to be optimized would be:

(22) $$\prod_{m=1}^{M}\frac{\sigma_{m}^{2}}{\sigma_{W}^{2}}.$$

However, to prevent very large values and overflow problems during optimization, the log function, not changing the convexity properties, is first applied to (22). Furthermore, to transform the measure provided by the FIM into a measure of the (weighted) dimensionality of the actually reconstructable unknown space in the same way as before, then (22) is further normalized by its value when only a single data component is exploited. Therefore, the functional to be optimized amounts to:

(23) $$\Phi^{\left(3\right)}\left({\underline{r}}_{1},\ldots ,{\underline{r}}_{N}\right)=\sum_{m=1}^{M}\left[\log\left(\sigma_{m}^{2}\right)-\log\left(\sigma_{1}^{2}\right)\right].$$

The considerations done for $\Phi^{\left(1\right)}$ and $\Phi^{\left(2\right)}$ apply also to $\Phi^{\left(3\right)}$.

### 3.7. Determining *N* and Optimizing the Functionals

**Figure 2:**
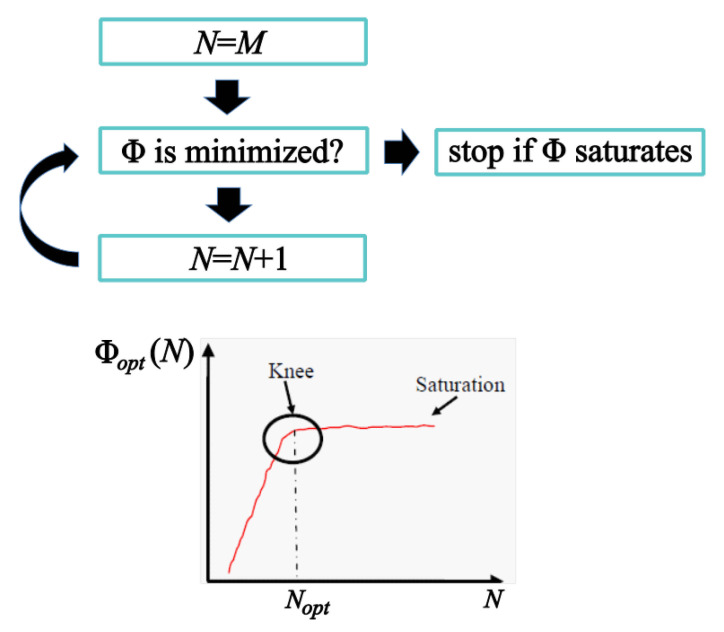
Flow chart for the determination of the number *N* of sampling points.

Concerning the determination of the number *N* of sampling points, an iterative approach is adopted, see Figure 2. Starting with *N* equal to *M*, *N* is progressively increased by one unit at each step, namely, a new sample is added, and the spatial distribution of the NF samples optimizing $\Phi^{\left(i\right)}$ is determined, reaching the optimum functional value $\Phi_{opt}^{\left(i\right)}\left(N\right)$. Since the adopted functional provides an information measure, the $\Phi_{opt}^{\left(i\right)}\left(N\right)$’s are expected to be essentially increasing functions of *N*. However, a saturation behavior is expected since, beyond a certain threshold $N_{0}$, even by adding samples, further information is not gathered. This value $N_{0}$ is the optimal (minimum) number of samples to retrieve as much information as possible about $E_{a}$ from the field samples.

Regarding the maximization of the $\Phi^{\left(i\right)}$’s, the optimization technique must be efficient, since the computational burden can be significant, due to the large number of unknowns involved, and effective, since we are interested in the global maximum for $\Phi^{\left(i\right)}$, and the use of local optimization techniques can be stuck into local optima. In order to simultaneously face both these issues, we resort to scheme in [14,15,16,17,18]. It should be mentioned that, in [18], a global optimizer was used. However, our experience is that, typically, local optimization, which has been used throughout this paper, returns already satisfactory results.

## 4. Numerical Results

We present now two test cases to discuss the behavior of SVO when using the three considered information measures, namely, Shannon number, mutual information, and Fisher information. The analysis is performed on numerically generated data to achieve full control of the tests and make a fair comparison among the metrics. In the first test case, say case A, a horn antenna is examined, while, in the second one, say case B, the AUT is a broadside array. In both cases, the radiated field has been obtained numerically by using the commercial software Altair FEKO. The SVO results will be compared against a reference provided by a regularized TSVD inversion exploiting the same PSWFs-based aperture field representation and a standard λ/2 sampling on *S*.

### 4.1. Horn Antenna

In case A, a 4λ×3λ sized aperture working at 10 GHz and represented with 8×6 visible PSWFs is considered while the domain *S* corresponds to a 30λ×30λ sized NF surface located at d=7λ. The aperture size will be larger than the physical horn aperture to incorporate the decay to zero region of the horn field on the z=0 plane.

The numerically generated data have been corrupted with noise with a Signal to Noise Ratio (SNR) of 35dB and the value of $\sigma_{U}^{2}/\sigma_{W}^{2}$ has been fixed consequently.

**Figure 3:**
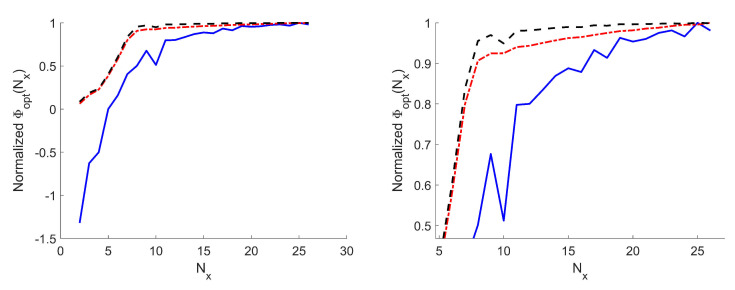
Case A. $\Phi_{opt}^{\left(i\right)}\left(N_{x}\right)$ curves—The $\Phi_{opt}^{\left(1\right)}\left(N_{x}\right)$ curve is reported in a log scale—all curves are normalized to their respective maxima. Blue solid line: Shannon number. Black dashed line: Mutual information. Red dash-dotted line: Fisher information. (**Left**) full curves. (**Right**) zoom around the knees.

Thanks to the fact that, using the approach in [14,15,16,17,18], the actual sampling grid is obtained as a distortion of a Cartesian grid, to simplify the determination of the optimal value of *N*, we have set $N=N_{x}\times N_{y}$, with $N_{x}$ and $N_{y}$ the number of samples along the *x*- and *y*-axes, respectively, while the ratio $N_{x}/N_{y}$ has been set equal to the ratio between the PSWFs needed to expand $E_{a}$ along *x* and *y*, namely 8/6. In this way, $\Phi_{opt}^{\left(i\right)}=\Phi_{opt}^{\left(i\right)}\left(N_{x}\right)$. Figure 3 illustrates the behavior of $\Phi_{opt}^{\left(i\right)}\left(N_{x}\right)$ in order to proceed to the choice of $N_{opt}$ for case A. As seen in Figure 3, both $\Phi_{opt}^{\left(2\right)}$ and $\Phi_{opt}^{\left(3\right)}$ are quite regular, while $\Phi_{opt}^{\left(2\right)}$ is less smooth due to the local minima issue. For the three metrics, saturation is reached approximately for $N_{x}=19$. In other words, by increasing $N_{x}$ beyond 19, none of $\Phi_{opt}^{\left(i\right)}$, i=1,2,3, significantly increases, meaning that the maximum amount of collectable information is reached with $N_{x}=19$.

**Figure 4:**
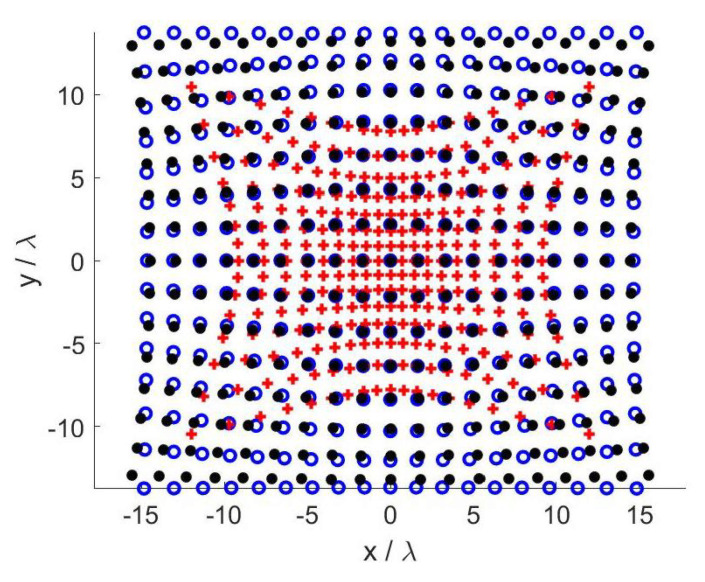
Case A—Optimized sampling points. Blue circles: Shannon number. Black dots: Mutual information. Red pluses: Fisher information.

Figure 4 depicts the optimized sampling points for the three SVO applications in case A.

**Figure 5:**
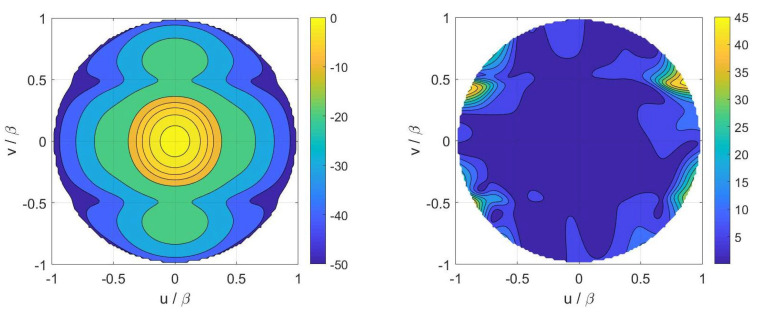
Case A—(**Left**) contour plot of the reference FFP. (**Right**) percentage error of the FFP retrieved in the Shannon number case.

**Figure 6:**
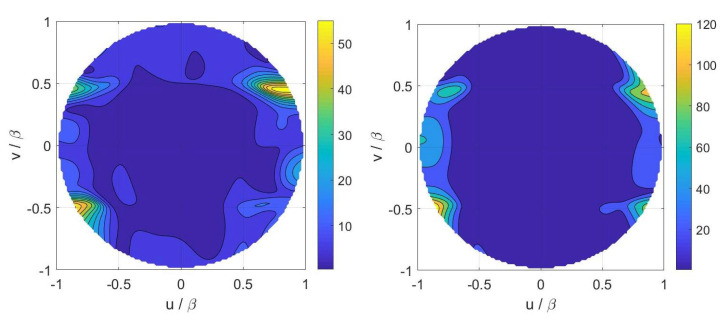
Case A—(**Left**) percentage error of the FFP (Far Field Pattern) retrieved in the mutual information case. (**Right**) percentage error of the FFP retrieved in the Fisher information case.

**Figure 7:**
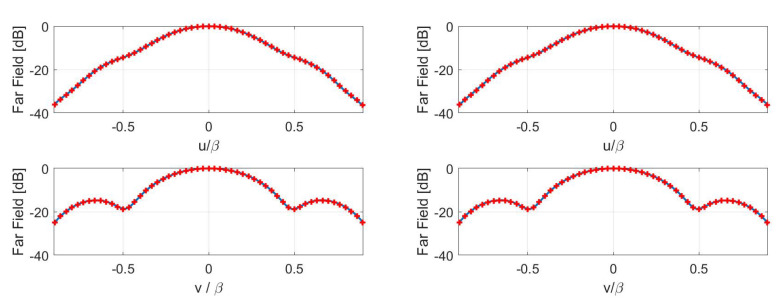
Case A—Cuts, along the *u* and *v* planes, of the reference (red pluses) and retrieved (blue solid line) FFP. (**Left**) Shannon number. (**Right**) mutual information.

**Figure 8:**
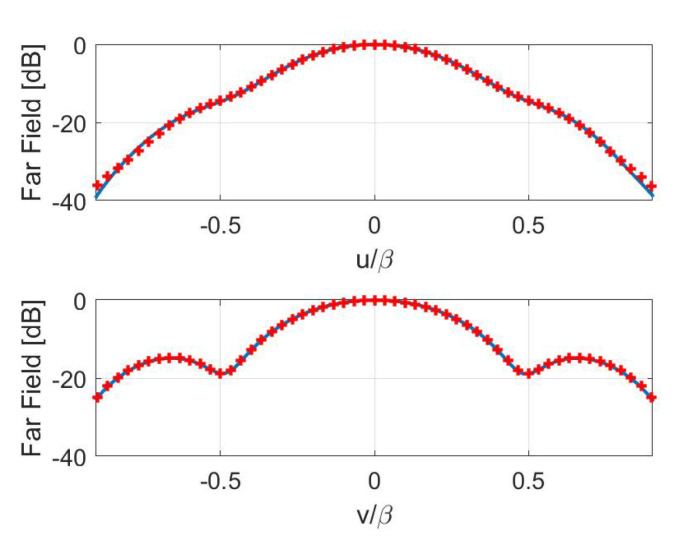
Case A—Cuts, along the *u* and *v* planes, of the reference (red pluses) and retrieved (blue solid line) FFP. Fisher information.

Finally, inversions for case A have been performed by considering the numerical field radiated by a horn antenna having a physical aperture size equal to 3λ×2λ. In particular, Figure 5 displays the contour plot of the reference Far Field Pattern (FFP) in the (u,v) plane along with the percentage errors achieved in the Shannon number case while Figure 6 shows those achieved in the mutual information and Fisher information cases. Furthermore, Figure 7 and Figure 8 report cuts, along the spectral *u* and *v* axes, of the retrieved FFPs for the three metrics superimposed to the numerical reference. The cuts have been reported for |u|,|v|≤0.9β due to the limitations of the aperture model. As it can be seen, satisfactory results are obtained in all the considered cases. Furthermore, the performance is very similar notwithstanding the different distribution of the samples. This should not be surprising since this behavior is related to the analytical properties of the involved fields.

### 4.2. Array

In case B, a 6λ×5λ sized aperture working at 2.4 GHz with an aperture field represented with 12×10 visible PSWFs is considered. The domain *S* is 30λ×30λ sized and located at d=7λ.

In addition, for this case, the numerically generated data have been corrupted with noise with a SNR of 35dB and the value of $\sigma_{U}^{2}/\sigma_{W}^{2}$ has been fixed consequently.

**Figure 9:**
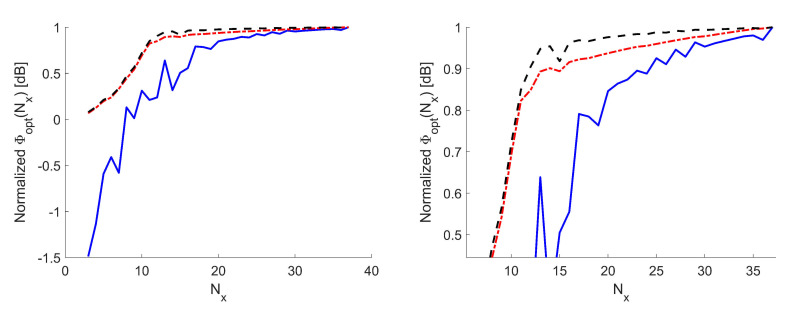
Case B. $\Phi_{opt}^{\left(i\right)}\left(N_{x}\right)$ curves. The $\Phi_{opt}^{\left(1\right)}\left(N_{x}\right)$ curve is reported in a log scale—all the curves are normalized to their respective maxima. Blue solid line: Shannon number. Black dashed line: Mutual information. Red dash-dotted line: Fisher information. (**Left**) full curves. (**Right**) zoom around the knees.

**Figure 10:**
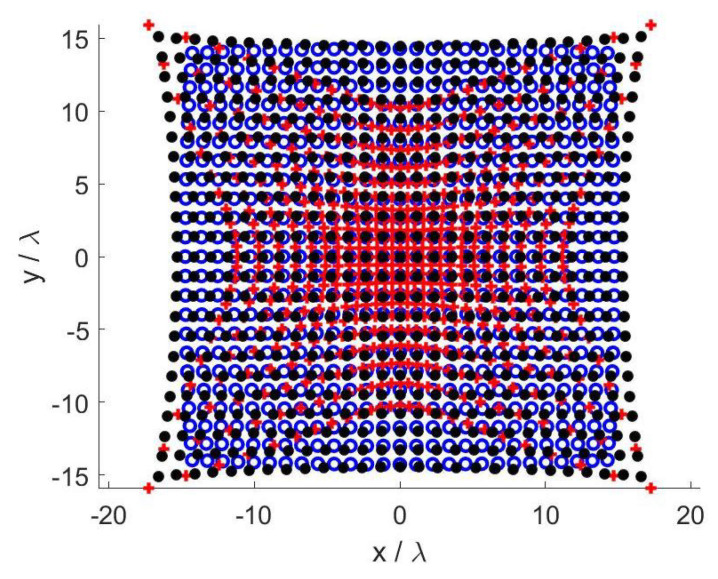
Case B—Optimized sampling points. Blue circles: Shannon number. Black dots: Mutual information. Red pluses: Fisher information.

Again, we have set $N=N_{x}\times N_{y}$ and the ratio $N_{x}/N_{y}$ has been fixed equal to 12/10 according to the number of PSWFs needed to represent the aperture field along *x* and *y*. In Figure 9, the choice of *N* for the three cases is reported. As before, $\Phi_{opt}^{\left(2\right)}$ and $\Phi_{opt}^{\left(3\right)}$ are quite regular, while $\Phi_{opt}^{\left(1\right)}$ shows small oscillations. For all the three metrics, the adopted value for $N_{x}$ has been 27. In other words, by increasing $N_{x}$ beyond 27, none of $\Phi_{opt}^{\left(i\right)}$, i=1,2,3, significantly increases thus meaning that the maximum amount of collectable information is reached with $N_{x}=27$. The corresponding distributions of the measurement locations for the three variants are reported in Figure 10.

**Figure 11:**
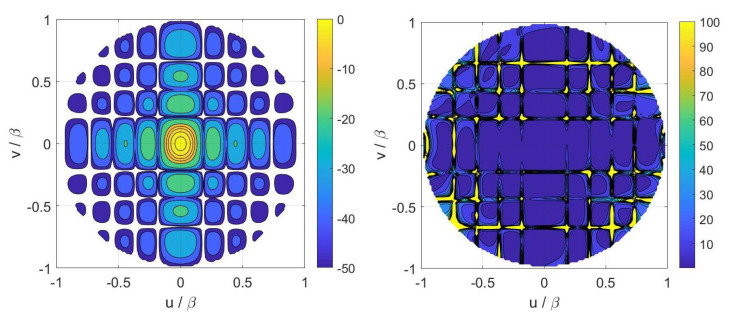
Case B—(**Left**) contour plot of the reference FFP. (**Right**) percentage error of the FFP retrieved in the Shannon number case.

**Figure 12:**
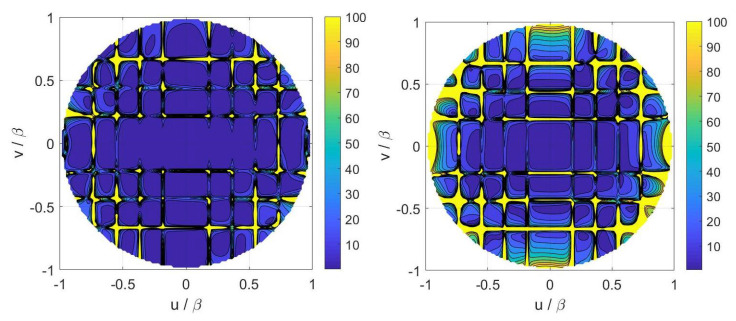
Case B—(**Left**) Percentage error of the FFP retrieved in the mutual information case. (**Right**) percentage error of the FFP retrieved in the Fisher information case.

**Figure 13:**
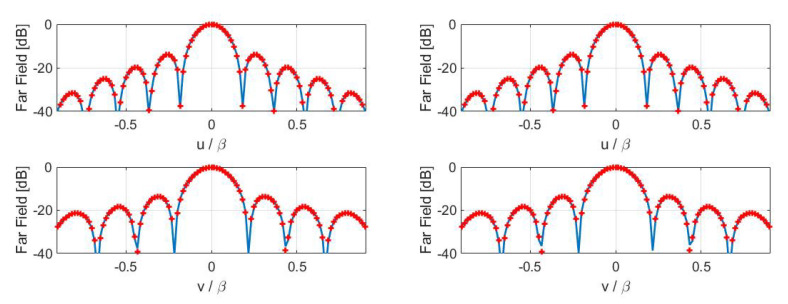
Case B—Cuts, along the *u* and *v* planes, of the reference (red pluses) and retrieved (blue solid line) FFP. (**Left**) Shannon number. (**Right**) mutual information.

**Figure 14:**
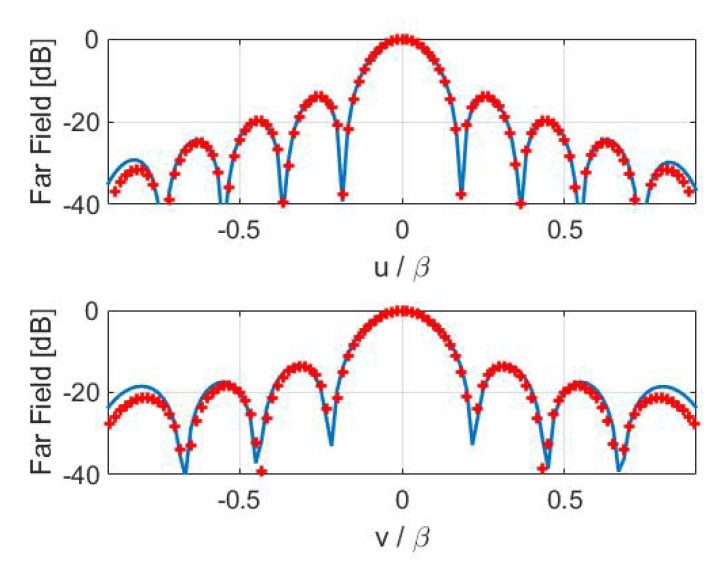
Case B—Cuts, along the *u* and *v* planes, of the reference (red pluses) and retrieved (blue solid line) FFP. Fisher information.

Concerning the inversions for case B, a broadside array of 11×9 elements with half-wavelength spacing has been simulated. The percentage reconstruction errors for the three approaches are shown in Figure 11, Figure 12, Figure 13 and Figure 14 as for case A. The results confirm that all the metrics have a satisfactory and comparable performance.

## 5. Conclusions and Future Developments

The SVO technique, when applied to a NFFF transformation problem, has the following features:

- It formulates the NFFF transformation problem as a linear inverse one;
- It uses an effective representation of the aperture field of the AUT (PSWFs);
- It determines the “optimal” near-field samples by maximizing the information acquired by the samples;
- The information measure acquired so far has been based on the Shannon information.

The challenge faced in the present paper was:

- To analyze the performance of SVO when different information metrics are considered.

We have therefore reconsidered the SVO approach for the characterization of antennas from NF data under different perspectives provided by different quality metrics. In particular, we also dealt with, apart from Shannon number, quality metrics provided by mutual information and Fisher information and numerically compared the performance of three SVO implementations.

In all the worked out cases, the number of exploited NF samples were the same and the achieved performance was essentially comparable although the optimized samples exhibited different distributions. This should not be a surprise due to the analytical properties of the radiated NF.

We explicitly mention that, in the authors’ experience, a similar performance achieved by Shannon number, mutual information, and Fisher information is not dependent of the particular test case under consideration, but it occurs uniformly over a wide range of parameters of the measurement configuration. We also underline that the SVO approach and the analysis herein contained applies to either individual radiating elements or more complex antenna systems and that SVO has been recently applied to tomographic problems in [35].

Future development of the present investigation are generalizing the approach to phaseless NFFF transformations [14,28] and considering the effect of correlated statistics of unknown and noise.

## Data Availability

Not applicable.
